# Perinatal outcome in anti-NMDAr encephalitis during pregnancy—a systematic review with individual patients’ data analysis

**DOI:** 10.1007/s10072-024-07448-1

**Published:** 2024-04-24

**Authors:** Giovanna Scorrano, Fedele Dono, Clarissa Corniello, Stefano Consoli, Giacomo Evangelista, Armando Di Ludovico, Francesco Chiarelli, Francesca Anzellotti, Angelo Di Iorio, Stefano L. Sensi

**Affiliations:** 1 Department of Pediatrics, “G. d’Annunzio” University of Chieti-Pescara, Chieti, Italy; 2 Department of Neuroscience, Imaging and Clinical Science, “G. d’Annunzio” University of Chieti-Pescara, Chieti, Italy; 3 Department of Aging Science, “G. d’Annunzio” University of Chieti-Pescara, Chieti, Italy; 4 Institute of Neurology, Epilepsy Center, “SS Annunziata” Hospital of Chieti, Chieti, Italy

**Keywords:** Autoimmune encephalitis, Pregnancy, Epilepsy, Fetal outcome, NMDAr antibody titer

## Abstract

**Introduction:**

Anti-N-methyl-D-aspartate receptor (NMDAr) antibody encephalitis is an autoimmune disorder characterized by synaptic NMDAr current disruption and receptor hypofunction, often affecting women during pregnancy. Clinical manifestations associated with anti-NMDAr encephalitis can occur both in the mother and fetus.

**Methods:**

We generated a systematic search of the literature to identify epidemiological, clinical, and serological data related to pregnant women with anti-NMDAr encephalitis and their children, analyzing the fetal outcomes. We examined the age and neurologic symptoms of the mothers, the presence of an underlying tumor, immunotherapies used during pregnancy, duration of the pregnancy, and type of delivery.

**Results:**

Data from 41 patients were extrapolated from the included studies. Spontaneous interruption of pregnancy, premature birth, and cesarean section were reported in pregnant women with NMDAr encephalitis. Several fetal and neonatal symptoms (e.g., movement disorders, spina bifida, poor sucking, respiratory distress, cardiac arrhythmias, infections, icterus, hypoglycemia, and low birth weight) depending on the mother’s serum anti-NR1 concentration were also reported.

**Conclusions:**

We characterized the outcomes of children born from mothers with anti-NMDAr encephalitis, analyzing the pivotal risk factors related to pregnancy and maternal disorder. Neuropsychiatric involvement seems strictly related to pathogenic NMDAr antibodies detected in maternal and/or neonatal serum.

These findings clarify a complex condition to manage, outlining the risks associated with pregnant women with anti-NMDAr encephalitis and also providing a concrete guide for therapeutic strategies to prevent potential harm to the fetus and the child’s neurodevelopment.

**Supplementary Information:**

The online version contains supplementary material available at 10.1007/s10072-024-07448-1.

## Introduction

Anti-N-methyl-D-aspartate receptor (NMDAr) autoimmune encephalitis (AE) is one of the most common causes of noninfectious encephalitis during pregnancy [1–3]. It is characterized by an autoimmune response against the NR1 subunit of NMDAr, which causes a reversible internalization of the receptor into neurons, leading to a more extended NMDAr channel opening and excessive synaptic and extra-synaptic NMDAr activation [4–6].

From the clinical point of view, the subacute onset of several neurological (e.g., cognitive decline, speech impairment, seizures, central hypoventilation, and movement disorders) and psychiatric (e.g., psychosis, anxiety, and depression) symptoms is recognized as diagnostic hallmarks. Furthermore, according to Graus’ criteria [7], laboratory (i.e., cerebral spinal fluid/serum specific auto-antibodies positivity) and radiological (i.e., mesial-temporal signal abnormalities in MRI T2 fluid-attenuated inversion recovery (FLAIR) images of the brain) findings can help the diagnostic process. Anti-NMDAr AE is frequently associated with an underlying tumor pathology, mostly ovarian teratoma, which detection is fundamental for treatment purposes.

Experimental and clinical evidence support the risk of early postnatal mortality and the increased prevalence of neurologic and systemic abnormalities in newborns delivered by mothers affected by anti-NMDAr AE during pregnancy. This phenomenon is partially related to the specific treatment employed for AE management (i.e., antiseizure medications and immunomodulatory drugs) as well as diagnostic interventions (i.e., computer tomography (CT) or magnetic resonance image (MRI) scans with contrast agents) whose teratogenic potential is already well documented. On the other hand, animal models have shown that maternal-to-fetal anti-NR1 auto antibodies transfer can be associated with a dose-dependent altered fetal neurodevelopment which may lead to growth retardation and impaired cognitive functions. Anti-N1 antibodies are an IgG class of antibodies that can cross the placental barrier from the 13th week of gestational age onwards.

This systematic review analyzed the available data on perinatal outcomes of newborns whose mothers have been affected by anti-NMDAr encephalitis during pregnancy. We also highlighted possible risk factors associated with increased newborns’ perinatal mortality and morbidity.

## Methods

### Searching strategy and review organization

We systematically reviewed the literature using the following search strategy: (“autoimmune encephalitis”/exp OR “autoimmune encephalitis”) AND (“fetal outcome”/exp OR “pregnancy”). The following electronic databases and data sources were systematically searched: MEDLINE (accessed through PubMed), Scopus, and Google Scholar. As per inclusion criteria, we evaluated all studies which (1) reported a confirmed diagnosis of anti-NMDAr encephalitis during pregnancy according to Graus’ criteria and (2) reported fetal and/or newborn outcomes. We included only papers written in English.

Results of this systematic review have been reported following the guidelines of the Preferred Reporting Items for Systematic Reviews and Meta-Analyses (PRISMA) statement. The quality of the included studies was assessed using the Newcastle–Ottawa Quality Assessment Scale (NOS). According to this scale, each study has been evaluated based on eight items, described as follows: (1) representativeness of the exposed cohort, (2) selection of the not exposed cohort, (3) ascertainment of exposure, (4) demonstration that outcome of interest was not present at the start of the study, (5) comparability of the cohorts included, (6) assessment of outcome, (7) adequate length of the follow-up, (8) adequacy of follow-up of cohorts. This score ranges from 0 to 9, and a quality score equal to or higher than three was considered acceptable.

### Data collection

The following demographic and clinical information about the mother have been collected: age, gestational age, history of epilepsy, comorbidities, neurologic symptoms at AE onset, seizure characteristics (seizure type), status epilepticus (SE) characteristics, presence of an underlying tumor, EEG features, magnetic resonance image (MRI) findings, immunomodulatory therapy, ASM administered (number, and type), and surgery procedures performed.

Data on stillbirth, type of delivery (vaginal or cesarean), Apgar score 1 and 5 min after delivery, neonatal symptoms, and NMDAr antibodies dosage at birth were collected.

The data were recorded within a specialized Excel spreadsheet.

### Statistics

Statistical analysis was performed on the final dataset containing all information pooled from the studies selected by our systematic review. Data were analyzed in IBM SPSS™; the normality of continuous data was checked via the Kolmogorov–Smirnov test. The Fisher chi-square test was employed to compare the perinatal outcome (born at term Vs preterm delivery; born at term Vs spontaneous abortion) according to maternal clinical and treatment features. Alpha level was set at 0.05 for statistical significance.

## Results

### Literature search

**Fig. 1:**
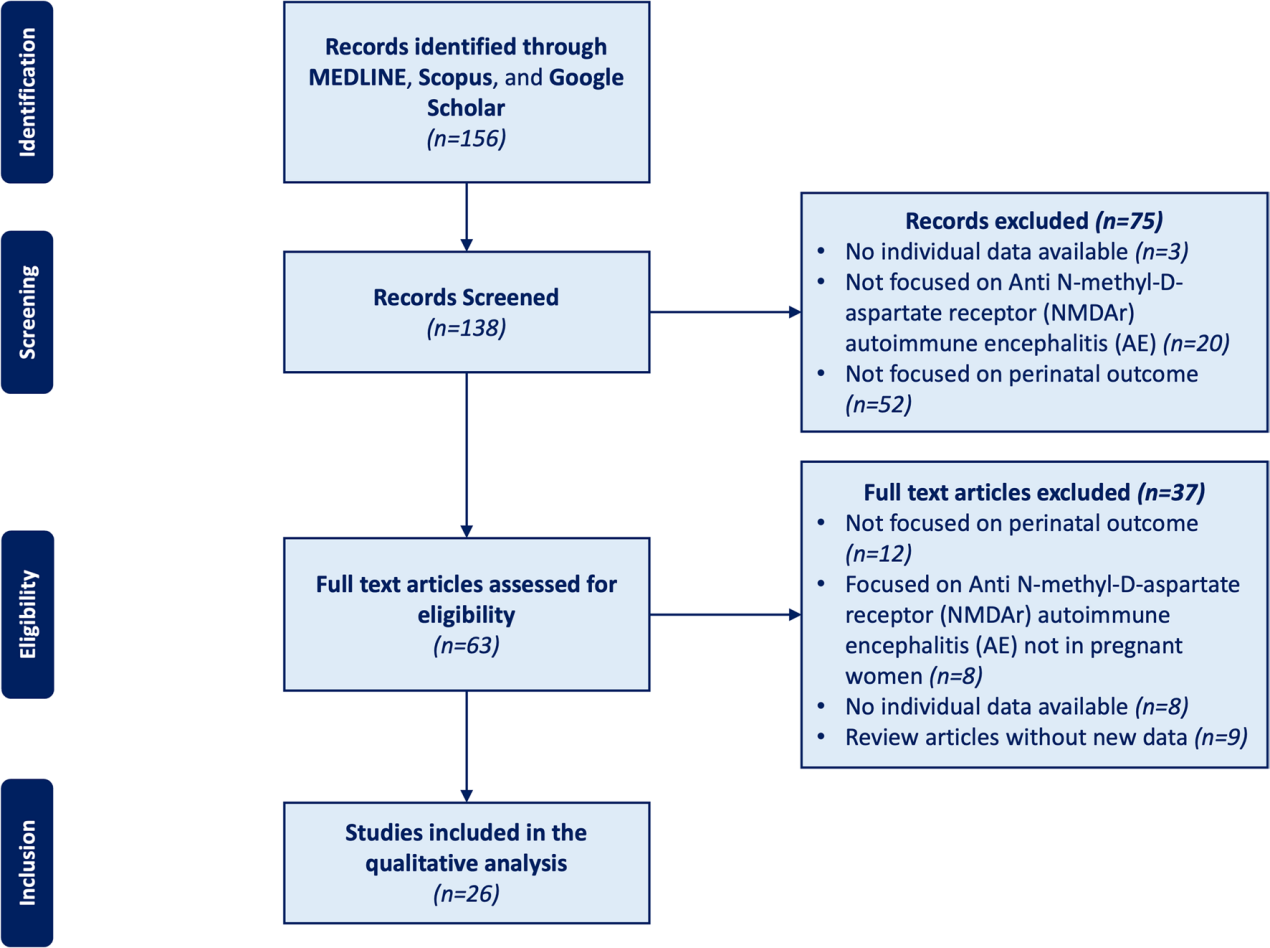
Flow chart of the article screening process. The Preferred Reporting Items for Systematic Reviews and Meta-Analyses diagram describes the search from the literature; 138 records were screened; from which, 26 articles were selected

The literature search reported above yielded 156 articles. Seventy-five abstracts were excluded because they did not focus on anti-NMDAr AE during pregnancy or perinatal outcome or did not report individual patients’ data. Of the 138 records screened, the full texts of 63 articles were reviewed for eligibility (Fig. 1). Thirty-seven articles initially considered for possible inclusion were eventually excluded (excluded articles with reasons for exclusion are reported in Fig. 1), and twenty-six were finally included in our review [8–33]. They included twenty-three case reports, and three case series (Table 1). According to the NOS evaluation, 13 articles were scored 5, 6 were scored 4, and 7 were scored 3 (Supp. Tab.1).

**Table 1:** Patients clinical and neuro-electrofisiological features

Study	Patients	Maternal Seizures type	SE during pregnancy	Oncologic evaluation	Delivery	Apgar (1,5 minutes)	IMT	ASMs	Surgery	Born pre-term	Newborns’ sympotms	Serum anti-NMDAr antibodies in newborns	Maternal presenting symptoms and comorbidities	Maternal additional therapies	Maternal EEG	Maternal Brain MRI
Sabrina Kalam et al. 2019 [8]	1	GTCS	No	Teratoma	SD	\	Iv CS, IVIg, PLEX	\	Tumor resection	Yes	\	\	\	\	Non-specific diffuse cortical dysfunction with no epileptiform activity	Amygdala hyperintensity bilaterally, more marked on the right
Kim J. et al. 2015 [9]	1	\	\	Teratoma	SI	\	IV CS, PLEX, RTX	\	Tumor resection	\	\	\	None	\	\	Normal
Mathis S. et al. 2015 [10]	1	GTCS	Yes	Negative	SD	10	IVIg, IV CS	CNZ, PHT, LMT	\	No	No symptoms	\	\	\	\	\
Lai Wan Chan et al. 2015 [11]	1	\	\	Teratoma	SI	\	CS, PLEX, RTX	\	Tumor resection	\	\	\	History of multiple suicide attempts	\	Bilateral rhythmic and semi-rhythmic delta activities (predominantly frontal)	T2-FLAIR Hyperintensities of right hippocampus and cerebellar parenchyma
Leah M. Lamale-Smith et al. 2015 [12]	1	Not specified seizure type	Yes	Negative	CSe	3,4	IVIg, CS, PLEX	LEV, TPM, Midazolam, PHT, CLB	Bilateral oophorectomy	Yes	Low birth weight, supraventricular tachycardia	Positive, 1:20	Depression and anxiety	\	Diffuse background slowing, right temporal discharges	Medial left temporal lobe and bilateral insula T2 Hyperintensities
Jagota P. et al. 2014 [13]	1	\	\	Negative	CSe	4,7	CS, IVIg	\	\	Yes	Intermittent episodes of continuous fine abnormal movements	Positive, 1:450	\	\	Diffuse slow waves with no epileptic discharges	\
Lu J. et al. 2015 [14]	1	No	No	Negative	SD	\	CS, IVIg	\	\	No	None	\	Visual hallucination, hyposexuality, speech disturbance	\	Normal	No acute intracranial processes
Magley J. et al. 2012 [15]	1	No	No	Negative	SD	8,9	CS, IVIg, PLEX	\	\	Yes	Torticollis, strabismus	\	Coreoathetosis, bradykinesia, weakness, depression	Clonazepam, sertraline, gabapentin, haloperidol, venlafaxine	Intermittent polymorphic bilateral frontal slowing	Abnormal T2 signal hyperintensity in bilateral caudate, globus pallidus and putamen
Kumar et al. 2010 [16]	1	GTCS	No	Teratoma	CSe	3,6	IVIg, IV CS	PHT, Lorazepam	Tumor resection	\	No symptoms	\	Headache, malaise, bizarre behavior, paranoid delusion	Not specified	Generalized slowing	Not specified
	1	GTCS	Yes	Teratoma	SI	\	IVIg	MDZ, CBZ, GBP	Tumor resection	\	\	\	Abnormal behavior, History of ovarian teratomas	Not specified	1 Hz spikes and slow activity in the frontal lobes	Not specified
	1	FS, GTCS	No	Negative	SD	8,9	CS	PB	\	No	No symptoms	\	Abnormal behavior	Not specified	Generalized high-amplitude slow activity	Normal
Shahani L., 2015 [17]	1	No	No	Negative	SD	\	Iv CS, PLEX	\	\	No	No symptoms	Negative	Bizarre behavior, paranoid delusion	\	Normal	Normal
McCarthy A. et al. 2012 [18]	1	No	No	Teratoma	CSe	\	IV CS, PLEX	\	Tumor resection	Yes	No symptoms	\	Urinary retention, constipation, new daily persistent headache	\	Diffuse slowing	Normal
Ito et al. 2010 [19]	1	GTCS	No	Negative	SD	\	CS	PB	\	No	No symptoms	\	Irritability and speech disturbances	\	Diffuse high-voltage slow waves	Normal
Xiao X. et al. 2017 [20]	1	GTCS	No	Negative	CSe	9	IVIg, IV CS	LZP, CNZ, CBZ, LEV, VPA	Wedge-shaped resection of bilateral ovaries (no oncological indication)	Yes	No symptoms	\	Psychiatric symptoms, visual and auditory hallucinations	\	Generalized seizures during sleep	Normal
Liu H. et al. 2021 [21]	1 (first pregnancy)	No	No	Negative	VA	\	IVIg, oral and IV CS	LMT	\	Yes	\	\	Psychiatric symptoms, oral-face-brachial dystonia	Olanzapine	Bilateral and diffuse persistent theta-delta slow waves	Hyperintense signal in the right hippocampus
	1 (second pregnancy)	No	No	Negative	VA	\	IVIg, IV CS	LEV, LMT	\	Yes	\	\	Psychiatric symptoms, epilepsy	\	Diffuse slow waves	Negative
Bastien J. et al. 2020 [22]	1	GTCS	No	Teratoma	CSe	\	IVIg, CS	LEV	Tumor resection	Yes	Respiratory distress, neuromuscular complications	Positive	Agitation, delusion, visual hallucinations, headache, oro-facial dyskinesia	Amoxicilline, Aciclovir	\	Mesio-temporal hyperintensity in T2 wighted sequences
	1	Not specified	No	Negative	CSe	-,9	CS, IVIg,	\	Tumor resection	Yes	No symptoms	\	Bulbar palsy, bilateral facial hypoesthesia, dysartria, bradychardia, agitation	\	\	Normal
	1	Not specified	No	Negative	CSe	\	CS, IVIg	\	\	No	Respiratory insufficiency	\	Oro-facial dyskinesia, memory problems	\	\	Bilateral mesio-temporal hyperintensity in T2 wighted sequences
	1	Temporal lobe seizure	No	Teratoma	CSe	-,10	IV CS, IVIg, PLEX, RTX	\	Tumor resection	Yes	No symptoms	\	Psychotic symptoms	\	\	Bilateral mesio-temporal hyperintensity in T2 wighted sequences
	1	GTCS	Yes	Negative	SD	-,10	CS, IVIg	LEV, Propofol	\	Yes	No symptoms	\	Behavioral changes, difficulties in speaking and reading	Amoxicilline, Aciclovir	\	Normal
	1	FAS	No	Teratoma	CSe	\	IV CS, IVIg	\	\	Yes	Low birth rate	\	Nausea, auditory hallucinations, catatonia, autonomic dysfunction, cardiac arrest	\	\	Normal
Kyu-On J. et al. 2020 [23]	1	GTCS, FS (motor seizures)	Yes	Negative	CSe	\	IVIg, CS, RTX	LEV, OXC, LCS, MDZ, CLB	\	Yes	No Symptoms	\	Headache	\	Continuous mixed, slow activity in the right temporal area. No epileptiform discharges	Right temporal and Insular cortices hyperintensity. ASL showed increased cerebral blood flow in the right insula and temporal area
Scorrano et al. 2023 [24]	1	GTCS	No	Negative	CSe	9	CS	LEV, LCS	\	No	Respiratory distress, hypoglycemia, jaundice, low birth weight, spina bifida	\	Psychiatric symptoms	\	\	Normal
Demma L. et al. 2017 [25]	1	GTCS	Yes (recurrent SE)	Teratoma	CSe	1,9	IV CS, IVIg, PLEX, RTX, Cyclophosphamide	LEV, LCS, PTH	Tumor resection	\	\	\	Anxiety, insomnia, hallucinations, fever	Antibiotics	\	Scattered white matter hyperdensities
Tailland M. et al. 2019 [26]	1	GTCS	No	Negative	CSe	\	IV CS, IVIg	LEV	\	No	No symptoms	\	Fever, left side hemiparesis, confusion, oro-facial dyskinesia,	\	\	Right perisilvian fissure and temporal lobe FLAIR hyperintensity.
Lu Y-T et al. 2016 [27]	1	GTCS	GCSE	Negative	CSe	\	\	OXC, VPA, PB	\	\	No symptoms	\	\	\	Left temporal ictal theta rhythm	Left mesial temporal hypersignal on FLAIR
	1	GTCS	GCSE	Negative	SD	\	\	PHT, VPA	\	Yes	No symptoms	\	\	\	Right central area focal slow	Superior sagittal sinus thrombosis with venous hemorrhagic infarction
	1	GTCS	GCSE	Negative	SI	\	\	LEV	\	\	No symptoms	\	\	\	Normal	Superior sagittal sinus thrombosis with venous hemorrhagic infarction
	1	Not specified	FSE	Negative	CSe	\	\	PHT, LEV, TPM, PB, VPA	\	\	Prematurity with complications	\	\	\	Left anterior quadrant rhythmic sharp waves	Mild hypersignal over left mesial temporal area
	1	GTCS	GCSE evolved to NCSE	Negative	SI	\	CS, PLEX	LEV, VPA, CLB, LMT	\	\	No symptoms	\	\	\	Ictal focal spikes over right frontocentral area	Bilateral frontoparietal area hypersignal on DWI; left mesial temporal hypersignal on FLAIR
	1	Not specified	FSE	Negative	SI	\	CS, IVIg,	VPA, PHT, CLB, LEV, PB, TPM	\	No	No symptoms	\	\	\	Bilateral independent ictal focal sharp delta activities	Hypersignal over bilateral medial temporal, left posterior insular and bilateral thalami on T2 imaging
	1	GTCS	GCSE evolved to NCSE	Teratoma	Not specified	\	CS, PLEX, AZA, Cyclophosphamide	VPA, LEV, TPM, CLB	No surgery	Yes	Not specified	\	\	\	Rhythmic bifrontal delta with superimposed sharp waves	Normal
Chourasia N.et al. 2018 [28]	1	Not specified	No	Teratoma	CSe	1,2	\	Lorazepam, LEV	Tumor resection	\	Intubation and mechanical ventilation, probable seizures	Positive, 1:320	History of anti-NMDA receptor encephalitis and unilateral oophorectomy	\	\	Diffuse cerebral edema
Ueda A. et al. 2017 [29]	1	No	No	Negative	CSe	\	IVIg, Iv CS, PLEX	\	\	\	No symptoms	Negative	Fever, oro-lingual-facial dyskinesia, choreoathetosis	\	Diffuse slowing	Normal
Zhang S. et al. 2020 [30]	1 (first pregnancy)	FAS	No	Negative	Not specified	\	Iv CS, IVIg	\	\	\	No symptoms	\	Visual hallucinations, delusions, systemic lupus erythematosus (SLE)	Hydroxychloroquine, prednisone	\	\
	1 (second pregnancy)	FAS	No	Negative	SI	\	IVIg, IV CS	LMT, LEV, VPA	\	\	\	\	Visual hallucinations, delusions, systemic lupus erythematosus (SLE)	Hydroxychloroquine, prednisone	Slow theta activity with an extreme delta brush pattern	Normal
Liao Z. et al. 2017 [31]	1	GTCS	No	Negative	CSe	9,10	CS, IVIg, PLEX	LEV, VPA	\	Yes	Low birth weight	\	Delirium, visual hallucinations, catatonia,	\	Paroxysmal middle- slow mixed wave.	Normal
Kokubun N.et al. 2016 [32]	1	No	No	Teratoma	VA	\	CS	\	Tumor resection	Yes	\	\	Involontary movement, hypoventilation, past history of a teratoma	\	\	\
Mizutamari E. et al. 2015 [33]	1	No	No	Teratoma	SD	\	PLEX, IVIg, CS	\	Right oophorectomy	No	No symptoms	Negative	Fever, headache, respiratory failure, nucal rigidity, history of left ovarian teratoma	\	\	\

*AZA* azathioprine, *CFSE* complex focal SE, *CLB* clobazam, *CLZ* clonazepam, oral or intravenous (IV) corticosteroid (CS), *CSe* Cesarean section, *FAS* focal aware seizure, *FS* focal seizure, *GTCS* Generalized tonic-clonic seizure, *GCSE* Generalized convulsive status epilepticus, *IVIg* Immunoglobulin intravenous, *LCS* lacosamide, *LEV* Levetiracetam, *LMT* lamotrigine, *MS* myoclonic seizure, *NCSE* non-convulsive status epilepticus, *NE* not evaluated, *OXC* oxcarbazepine, *PHB* phenobarbital, *PHT* phenytoin, *PLEX* plasma exchange, *PLEX* plasmapheresis, *pp* post-partum, *RTX* rituximab, *SD* spontaneous delivery, *SE* status epilepticus, *SI* spontaneous interruption, *SW* sharp-waves, *TPM* topiramate, *VA* voluntary abortion, *VPA* Valproic Acid

### Maternal demographics and clinical features

A literature search showed thirthy-nine pregnant women with a median age of 25 years (range, 16–36 years). Twenty-one patients (21/39, 53.8%) presented anti-NMDAr encephalitis onset within the first trimester of pregnancy, whereas 17 patients (18/39, 46.2%) during the second one. The most common presenting symptoms included abnormal behaviors, movement disorders, autonomic disturbance, and seizures. According to seizure type, nineteen patients (19/39, 48.7%) had tonic–clonic generalized seizures, and six patients (4/39, 10.3%) had only focal seizures. Thirteen patients (13/39, 33.3%) presented status epilepticus and required intensive care management. The oncological evaluation revealed the presence of ovarian teratoma in seventeen patients (14/39, 35.9%). EEG analysis results were reported in 26 patients (26/39, 66.7%) and documented slow activity and interictal epileptic abnormalities in 23 cases (23/39, 59%), whereas ictal discharges and extreme delta brush in three patients (3/39, 7.7%). Brain MRI was normal in all cases except for 21 patients (21/39, 58.3%), who showed cerebellum, hippocampus, bilateral amygdala, basal ganglia, and insular cortex hyperintensity in T2-weighted MRI scans.

Immunotherapy was administered to thirty-five patients (36/39, 92.3.%). Thirty-five patients (35/39, 89.7%) received high oral or EV corticosteroid therapy, 26 mothers (26/39, 66.7%) were treated with IGEV, 6 with third-line treatments (i.e. RTX, cyclophosphamide, and azathioprine) (6/39, 15.4%), and 14 with plasmapheresis (14/39, 35.9%). ASM was administered in 25 (25/39, 64.1%) patients, with levetiracetam (LEV) and phenytoin (PHT) being the most used.

Extensive demographics and clinical information are listed in Table 1.

### Perinatal outcomes

Data from 41 subjects in the perinatal period were evaluated. In two cases, the mothers suffered from a first episode of AE during a first pregnancy and a relapse during a second one. In 7 cases (7/41, 17.1%), a spontaneous interruption of the pregnancy was reported, whereas a voluntary interruption was reported in 3 (3/41, 7.3%) Of the 31 remaining alive subjects, 19 (19/31, 61.3%) were born from a cesarean section, and 10 (10/31, 32.3%) had a vaginal delivery. Nineteen subjects (18/31, 58.1%) experienced premature birth.

The APGAR score was available in 14 infants, showing a 5-min score within the normal range in 10. Serum anti-NMDAr antibody levels were tested in 7 cases (7/41, 17.1%) and found positive in 4. These patients showed perinatal complications, which mostly included neuromuscular and respiratory symptoms.

Above all the neurological manifestations reported, impaired neonatal reflexes (i.e., Moro, sucking, and grasping), cervical dystonia, strabismus, movement disorders, spina bifida, and seizures were the most reported. On the other hand, non-neurological symptoms mostly included respiratory depression, low birth weight, and supraventricular tachycardia 13.

Extensive information about perinatal outcomes is listed in Table 1.

### Statistical analysis of the pooled data

According to the data analysis of single patients, no differences were observed between mothers who complete their pregnancies and those who experienced a spontaneous interruption (Table 2). However, a trend towards a reduced risk of abortion was also observed in women treated with IVIg (*p* = 0.06).  In addition, a trend towards an increased risk of pre-term born was observed in mothers who underwent surgery procedures for teratoma removal (p=0.06) (Table 3).

**Table 2:** Frequency distribution of clinical features of patients enrolled according to spontaneous interruption of pregnancy. Data were reported as absolute number and relative percentage. Differences comparison between the two groups were assessed by Fisher chi-square test. *ASM* anti-seizure medication, *IVIg* intravenous Immunoglobulins, *PLEX* plasma exchanges, *RTX* rituximab

	Delivered pregnancies (*n*=31)	Spontaneous interruption (*n*=7)	*p*-value
*Teratoma*	10 (32.3)	3 (33.3)	0.95
*Oral corticosteroid*	16 (51.6)	3 (33.3)	0.33
*Intravenous corticosteroid*	10 (32.3)	4 (44.4)	0.49
*IVIg*	21 (67.7)	3 (33.3)	0.06
*PLEX*	9 (29)	2 (16.7)	0.69
*RTX*	3 (3.2)	2 (22.2)	0.31
*Seizure*	20 (64.5)	7 (77.8)	0.45
*Status epilepticus*	9 (29)	4 (44.4)	0.38
*ASM administration*	18 (58.1)	7 (77.8)	0.28
*ASM polytherapy (>2 ASM)*	8 (25.8)	4 (44.4)	0.28
*Surgery*	12 (38.7)	3 (33.3)	0.77

**Table 3:** Frequency distribution of clinical features of patients enrolled according to preterm birth. Data were reported as absolute number and relative percentage. Differences in comparison between the two groups were assessed by Fisher's chi-square test. *ASM* anti-seizure medication, *IVIg* intravenous Immunoglobulins, *PLEX* plasma exchanges), *RTX* rituximab

	Full term (*n*=10)	Preterm (*n*=18)	*p*-value
*Teratoma*	1 (10)	7 (38.9)	0.10
*Oral corticosteroid*	7 (70)	10 (55.6)	0.45
*Intravenous corticosteroid*	3 (30)	7 (38.9)	0.63
*IVIg*	6 (60)	14 (77.8)	0.31
*PEX*	2 (20)	7 (38.9)	0.30
*RTX*	0 (0.0)	1 (5.6)	NA
*Seizure*	5 (50)	11 (61.1)	0.56
*Status epilepticus*	2 (20)	5 (27.8)	0.64
*ASM administration*	6 (60)	10 (55.6)	0.81
*ASM polytherapy (> 2 ASM)*	2 (20)	4 (22.2)	0.89
*Surgery*	1 (10)	8 (44.4)	0.06

## Discussion

**Fig. 2:**
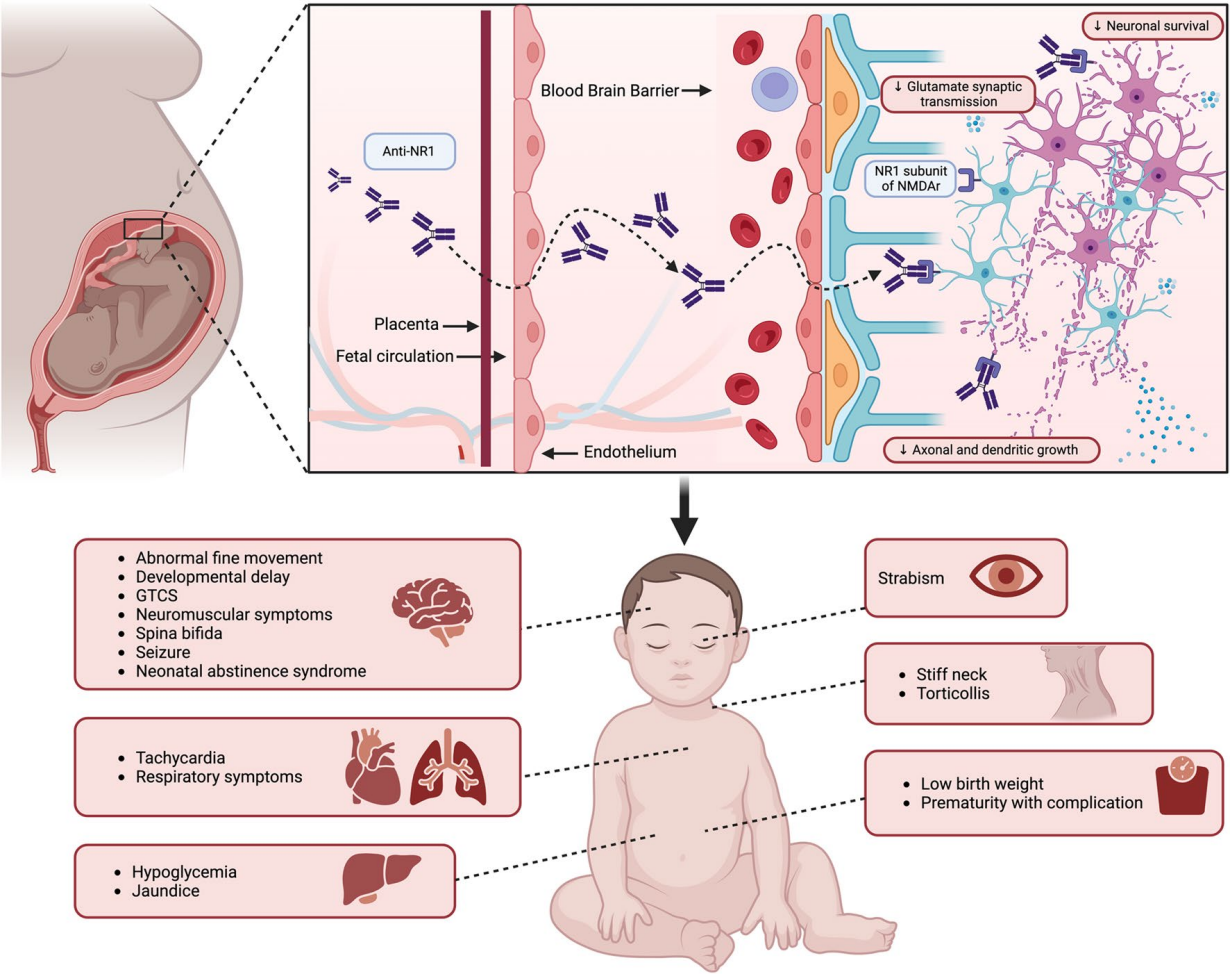
Clinical manifestations in newborns’ delivered by mothers suffering from anti-NMDAr encephalitis

Anti-NMDAr encephalitis is the most frequent autoimmune encephalitis during pregnancy [34]. According to our data, in the perinatal period, newborns delivered by mothers suffering from anti-NMDAr AE may show neurological (i.e., non-finalistic limb movements, cervical dystonia, strabismus, spina bifida, impaired Moro reflexes, poor sucking and grasping) as well as non-neurological (i.e., respiratory distress, neonatal infection, icterus, hypoglycemia, low birth weight, and supraventricular tachycardia) sequelae (Fig. 2). All individuals developing perinatal symptoms presented positive serum NMDAr antibodies [12, 13, 22, 28]. This evidence supports the notion of a harmful maternal-to-fetal NR1 autoantibody transfer extensively described in preclinical models. However, further concurrent factors should be explored as a putative cause of newborns’ perinatal symptoms onset. In fact, several therapeutic interventions largely employed in AE management such as ASM and immunomodulant therapies (IMT) are associated with a great risk of perinatal complications.

According to the literature, ASM exposure during pregnancy may increase the rate of preterm birth, intrauterine growth restriction, low Apgar score, neonatal hypoglycemia and sepsis, respiratory distress, major congenital malformations (MCMs), and/or cognitive-behavioral impairment [35]. Specifically, some ASM like valproate acid (VPA), phenobarbital (PB), phenytoin (PHT), carbamazepine (CBZ), and topiramate (TPM) have been labeled as the most dangerous in terms of fetal harm. Thus, their use during pregnancy should be avoided. On the other hand, lamotrigine (LMT) and LEV seems to be associated with a very low rate of major congenital malformations (MCMs) and perinatal distress [36, 37]. Surprisingly, according to our results, newborns exposed to VPA during pregnancy mostly presented normal Apgar score, a low rate of miscarriage (3/9, 33.3%), and prematurity with complications (1/9, 11%). However, this data should be interpreted with caution in light of publication and reporting biases.

A solid set of evidence indicates that IMT may increase perinatal disorders in newborns [34]. Even though first-line IMT (i.e., corticosteroids, plasma exchange, and intravenous immunoglobulin) seem to be safe, second-line IMT (i.e., azathioprine, mycophenolate mofetil, cyclophosphamide, and rituximab) should be used with caution given the potential harmful profile towards fetal and newborns’ health. In line with this evidence, our study did not revealed a significantly increased risk of spontaneous pregnancy interruption in mothers who received treatment with RTX. RTX is a chimeric anti-CD20 monoclonal antibody that leads to depletion of B cells in humans, with consequent hypogammaglobulinemia. RTX can cross the placental barrier, and its use during pregnancy has been associated with neonatal transient lymphopenia and decreased gamma globulin levels.

A potential increase of pre-term birth was also described for women with ovarian teratoma who underwent subsequent surgical treatment. According to the literature, pregnant women suffering from cancer generally show an increased risk of abortion (i.e., 10% higher) than the general population. Furthermore, large epidemiological studies have shown that non-obstetric surgery in pregnant patients is associated with small, but real, increases in the risks of stillbirth, preterm delivery, and the need for cesarean section. This is mainly related to the anesthesia risk, the pre-operatory imaging, the development of changes in fetal hemodynamics, and the fetal surgical stress, still largely unknown [38–42]. However, a fetal monitoring during surgery, anesthesia between 4 and 20 gestational weeks, a regular patient follow-up with high-resolution ultrasonography, and attention to clinical symptoms and other signs were associated with a relatively safe non-obstetric surgery [38–42].

## Conclusions

The management of pregnancy in women with anti-NMDAr encephalitis remains challenging. Our study depicted the potential outcomes of children born from mothers suffering from anti-NMDAr encephalitis and analyzed risk factors related to pregnancy and maternal disorders. To prevent complications that could harm the mother and the child, a personalized management should be enforced, targeting potential fetal risks related to anti-NMDAr encephalitis, autoantibodies, and therapy administered during pregnancy.

### Supplementary Information

Below is the link to the electronic supplementary material.

Supplementary file1 (DOCX 22.8 KB)

## Data Availability

The data that support the findings of this study are available from the corresponding author upon request.
