# Supplementary material for: Perinatal outcome in anti-NMDAr encephalitis during pregnancy—a systematic review with individual patients’ data analysis
Source: Neurol Sci. 2024 Apr 24;45(9):4211–27. doi: 10.1007/s10072-024-07448-1 (PMC11306544; doi:10.1007/s10072-024-07448-1)
Supplement: Supplementary file 1 — Supplementary file1 (DOCX 22.8 KB) [file 10072_2024_7448_MOESM1_ESM.docx]

| **Reference** | **Representativeness of the exposed cohort** | **Selection of the non exposed cohort** | **Ascertainment of exposure** | **Demonstration that outcome of interest was not present at start of study** | **Comparability of the cohorts included** | **Assessment of outcome** | **Was follow-up long enough for outcomes to occur?** | **Adequacy of follow up of cohorts** | **Total score** |
| --- | --- | --- | --- | --- | --- | --- | --- | --- | --- |
| Sabrina Kalam et al, 2019 | * | - | * | * | - | - | * | - | **** |
| Kim J. et al, 2015 | * | - | * | * | - | - | * | - | **** |
| Mathis S. et al, 2015 | * | - | * | * | - | * | * | - | ***** |
| Lai Wan Chan et al, 2015 | * | - | * | * | - | - | * | - | **** |
| Leah M. Lamale-Smith et al, 2015 | * | - | * | * | - | * | * | - | ***** |
| Jagota P. et al, 2014 | * | - | * | * | - | * | * | - | ***** |
| Lu J. et al, 2015 | * | - | * | * | - | - | - | - | *** |
| Magley J. et al, 2012 | * | - | * | * | - | * | * | - | ***** |
| Kumar et al., 2010 | * | - | * | * | - | * | * | - | ***** |
| Shahani L., 2015 | * | - | * | * | - | * | * | - | ***** |
| McCarthy A. et al., 2012 | * | - | * | * | - | * | * | - | ***** |
| Ito et al., 2010 | * | - | * | * | - | - | - | - | *** |
| Xiao X. et al., 2017 | * | - | * | * | - | * | - | - | **** |
| Liu H. et al, 2021 | * | - | * | * | - | - | * | - | **** |
| Bastien J. et al, 2020 | * | - | * | * | - | * | - | - | **** |
| Kyu-On J. et al, 2020 | * | - | * | * | - | * | * | - | ***** |
| Scorrano et al, | * | - | * | * | - | * | * | - | ***** |
| Demma L. et al. | * | - | * | * | - | - | - | - | *** |
| Tailland M. et al. | * | - | * | * | - | * | * | - | ***** |
| Lu Y-T et al. | * | - | * | * | - | - | - | - | *** |
| Chourasia N.et al, 2018 | * | - | * | * | - | * | * | - | ***** |
| Ueda A. et al, 2017 | * | - | * | * | - | * | * | - | ***** |
| Zhang S. et al., 2020 | * | - | * | * | - | - | - | - | *** |
| Liao Z. et al, 2017 | * | - | * | * | - | - | - | - | *** |
| Kokubun N.et al, 2016 | * | - | * | * | - | - | - | - | *** |
| Mizutamari E. et al, 2015 | * | - | * | * | - | * | * | - | ***** |

**Supp.Tab.1** Newcastle-Ottawa Quality Assessment Scale single paper score.
